# Oncogene addiction: resetting the safety switch?

**DOI:** 10.18632/oncotarget.2474

**Published:** 2014-09-20

**Authors:** Yulin Li, Peter S. Choi, Dean W. Felsher

**Affiliations:** Division of Oncology, Departments of Medicine and Pathology, Stanford University, Stanford, CA

The term “oncogene addiction” was first coined by Dr. Bernard Weinstein to describe the exquisite dependency of tumor cells on the expression of specific oncogenes for their relentless proliferation and survival [1]. Since cancers generally have accumulated multiple genetic and epigenetic abnormalities, it is surprising that they can remain dependent on any particular oncogenic driver. This “Achilles’ heel” of cancer has been widely exploited by targeted therapy of many human cancers, such as imatinib for BCR-ABL-driven leukemia, crizotinib for leukemia with FLT3-ITD mutations, gefitinib for lung adenocarcinoma with EGFR mutations, and vemurafenib for melanomas with B-RAF mutations. However, the mechanism of oncogene addiction is not clear.

Several non-mutually exclusive mechanisms for oncogene addiction have been proposed, such as synthetic lethality, genetic streamlining, oncogenic shock, and the safety switch model. In the synthetic lethality model, some mutations that develop during cancer evolution are either neutral or adaptive only in the presence of the driver oncogene [2]. However, these same mutations are deleterious to the cancer cells in the absence of the driver oncogene, rendering the cancer cells unfit for survival. In the genetic streamlining model, the cancer cells are rewired by the dominant oncogenic driver and lose the cellular functions that are not essential for survival and proliferation [3]. Thus, the tumors will collapse once the dominant signaling pathway upon which cancer cells are highly dependent is suppressed. The oncogenic shock model posits that there is a differential decay of the pro-survival and pro- apoptotic signals upon the inactivation of an oncogene. This differential decay results in a vulnerable window, causing the cell to irreversibly undergo apoptosis [4]. Previously, we also proposed the cellular safety switch model. We suggested that the inactivation of the driver oncogene restores the normal cellular safety switch, and thus leads to proliferative arrest, apoptosis, and/or cellular senescence [5, 6]. However, a molecular mechanism has not been elucidated for any of these models.

Now we have uncovered the molecular mechanism underlying the cellular safety switch model in MYC- induced tumors [7]. MYC inactivation is associated with the loss of many of the hallmark features of tumorigenesis and results in proliferative arrest, apoptosis, differentiation, and senescence, as well as the shutdown of angiogenesis. These phenotypes induced by MYC inactivation are surprisingly similar to those resulting from the loss in function of *miR-17-92*, a MYC target gene known to regulate multiple aspects of tumorigenesis, such as proliferation, survival, and angiogenesis.

Therefore, we speculated that *miR-17-92* could mediate MYC oncogene addiction. Indeed, we found *miR-17-92* expression rescued many of the phenotypes associated with MYC inactivation. Furthermore, we discovered that *miR-17-92* can target several histone modifiers, such as Sin3 transcription regulator family member B (Sin3b), high mobility group box transcription factor 1 (Hbp1), suppressor of variegation 4–20 homolog 1 (Suv420h1), and B cell translocation gene 1 (Btg1), as well as the apoptosis mediator Bcl2-like 11 (Bcl2l11, also known as Bim). Sin3b and Hbp1 coordinately recruit histone deacetylases to downregulate the expression of proliferation-related genes, resulting in cell cycle exit and senescence. Suv420h1 can regulate chromatin compaction and senescence by methylating histone H4 lysine 20. Btg1 can also regulate chromatin state, senescence, and differentiation by activating protein arginine methyltransferase 1 (Prmt1) to methylate histone H4 arginine 3. Thus, through coordinately regulating multiple epigenetic programs, these histone modifiers induce proliferative arrest and senescence upon MYC inactivation. By modulating the expression of these histone modifiers and the apoptosis regulator, Bim, *miR-17-92* can block the normal cellular safety switch and sustain the neoplastic state of MYC- driven tumors. Upon the inactivation of MYC, the downregulation of *miR-17-92* results in the induction of these target genes, which can drive senescence, apoptosis, and differentiation. Hence, MYC inactivation appears to reset the cellular safety switch and results in tumor collapse [7].

Although we observed our results in MYC-induced tumors, we speculate that other oncogenic drivers are likely to operate through similar if not identical mechanisms. For example, we have observed that suppression of RAS or BCR-ABL similarly activates senescence and apoptosis programs. This safety switch model supported by our experimental observations provides a possible general mechanistic explanation for the phenomenon of oncogene addiction.
